# Soil salinization accelerates microbiome stabilization in iterative selections for plant performance

**DOI:** 10.1111/nph.17774

**Published:** 2021-10-26

**Authors:** William L. King, Laura M. Kaminsky, Maria Gannett, Grant L. Thompson, Jenny Kao‐Kniffin, Terrence H. Bell

**Affiliations:** ^1^ Department of Plant Pathology and Environmental Microbiology The Pennsylvania State University University Park PA 16802 USA; ^2^ School of Integrative Plant Science Cornell University Ithaca NY 14853 USA; ^3^ Department of Horticulture Iowa State University Ames IA 50011 USA

**Keywords:** artificial selection, microbiome breeding, phenotype selection, rhizosphere, root microbiome, soil salinity

## Abstract

- Climate change‐related soil salinization increases plant stress and decreases productivity. Soil microorganisms are thought to reduce salt stress through multiple mechanisms, so diverse assemblages could improve plant growth under such conditions. Previous studies have shown that microbiome selection can promote desired plant phenotypes, but with high variability. We hypothesized that microbiome selection would be more consistent in saline soils by increasing potential benefits to the plants.
- In both salt‐amended and untreated soils, we transferred forward *Brassica rapa* root microbiomes (from high‐biomass or randomly selected pots) across six planting generations while assessing bacterial (16S rRNA) and fungal (ITS) composition in detail. Uniquely, we included an add‐back control (re‐adding initial frozen soil microbiome) as a within‐generation reference for microbiome and plant phenotype selection.
- We observed inconsistent effects of microbiome selection on plant biomass across generations, but microbial composition consistently diverged from the add‐back control. Although salt amendment strongly impacted microbial composition, it did not increase the predictability of microbiome effects on plant phenotype, but it did increase the rate at which microbiome selection plateaued.
- These data highlight a disconnect in the trajectories of microbiomes and plant phenotypes during microbiome selection, emphasizing the role of standard controls to explain microbiome selection outcomes.

## Introduction

Global climate change is increasing the severity and frequency of environmental perturbations. In addition to increasing temperatures, climate change is reshaping habitat space, through processes such as ocean acidification and soil salinization, threatening both biodiversity and ecosystem functioning (Malhi *et al*., 2020; Eswar *et al*., 2021). Climate‐driven soil salinization, driven by a combination of altered rainfall patterns, precipitation rates, and rising sea‐level, is a major problem for both natural and agricultural systems and exacerbates salinity issues from intensive land‐usage (for example, irrigation water and the groundwater table) (Eswar *et al*., 2021). Salinity stress significantly impairs plant physiology and development (Isayenkov & Maathuis, 2019), but soil microorganisms are thought to be able to mitigate some negative impacts of soil salinity on plants (Otlewska *et al*., 2020). Plants can modify and even increase root exudation and rhizodeposits under stressful conditions (Karst *et al*., 2017; Preece *et al*., 2018; Zhalnina *et al*., 2018; Williams & de Vries, 2020), which may allow them to influence recruitment of a beneficial rhizosphere microbiome in the presence of salt. Although various microbes have been implicated in supporting plant growth under saline conditions (Kumar *et al*., 2020; Xiong *et al*., 2020), it is not clear that any one taxon will have outsized impacts. Rather, there are several mechanisms by which microbes are thought to enhance plant growth in saline soils, including nutrient solubilization, ethylene reduction, and exopolysaccharide production (Kumar *et al*., 2020). As such, plant growth promotion likely results from the additive contributions of many taxa within the soil microbiome.

Aside from salt protection, key ecosystem services, like nutrient retention, plant nutrient acquisition, and plant pathogen defense, are provided largely, or in part, by soil microbiomes (Gordon *et al*., 2008; Mendes *et al*., 2011; Jacoby *et al*., 2017). In agricultural settings, effective microbial management may increase microbial contributions to these processes, reducing demands on chemical fertilizers and pesticides which can negatively impact human health, the environment, and plant–microbe relationships (Chambers *et al*., 1980; Streeter & Wong, 1988; Fox *et al*., 2007; Diaz & Rosenberg, 2008; van Diepen *et al*., 2010; Geiger *et al*., 2010; Damalas & Eleftherohorinos, 2011). Microbial products for agricultural plants have existed for over a century (Wiley, 1902), but coincident with recent successes in using microbiome transplants to treat human health conditions (Borody & Khoruts, 2012; Fuentes *et al*., 2014), many agricultural biotech companies have invested heavily into developing microbial products that stimulate plant growth (Fox, 2015; Waltz, 2017).

Some existing microbial products, such as mycorrhizal fungi and rhizobia, develop important one‐to‐one interactions with plants (Furseth *et al*., 2012; Hijri, 2016; Kaminsky *et al*., 2019) and can have large impacts on plant traits (van der Heijden *et al*., 2016). However, selecting for complex growth‐promoting assemblages of microbes may provide additional benefits by capturing microorganisms that fill multiple functional roles (e.g. fix nitrogen (N_2_) and solubilize phosphorus (P)), perform similar functions across diverse microenvironments within a given soil/root system (e.g. within low and high pH microsites), access different resources (e.g. metabolize different types of soil carbon (C)), or cooperatively execute plant growth‐promoting functions. Additionally, microbial assemblages are known to function differently when evolved in combination rather than in isolation (Lawrence *et al*., 2012; Fiegna *et al*., 2015; Ketola *et al*., 2016), making it difficult to predict the intermicrobial interactions and in‐soil phenotypes of microbiomes based solely on which taxa are present (Bell *et al*., 2019). As a result, selecting for beneficial soil microbial assemblages based on plant phenotypes (or other observable group traits) is an appealing concept.

In traditional plant breeding, progressive selection based on phenotype can enhance desired traits, even without an in‐depth understanding of a plant’s genetic makeup. However, the heritability of microbiome composition and/or functional attributes through plant phenotype‐guided selection may be entirely dissimilar, given that selection is imposed not on one individual, but many. Previous studies show that the directional selection of microbiome traits is plausible, but that trajectories are noisy. For example, selection for low CO_2_ emissions in microbiomes led to reductions in CO_2_, but with large rebounds observed (Blouin *et al*., 2015). Similar outcomes were observed when selection targeted plant phenotypes like flowering time and biomass (Swenson *et al*., 2000; Panke‐Buisse *et al*., 2015). These studies showed detailed changes in microbiome‐driven phenotypes across selection generations, but this was not paired with between‐generation changes in microbial composition. As a result, it is unclear whether phenotypic variability during microbiome selection is accompanied by microbiome variability, which was the expected driver of phenotypic change. Although shifts in plant phenotype are often noisy during microbiome selection, we expected that adding a soil stress would increase plant reliance on soil microorganisms, leading to more rapid and stable phenotype selection.

In this study, we aimed to determine how progressive selection of root‐associated microbiomes impacted plant phenotypes in both salt‐amended soils and soils with no added salt. We transferred microbiomes associated with the roots of mature *Brassica rapa* after 32 d of growth to new sterile soils, which were seeded with a consistent seed pool (no selection performed on plant genotype), and this was repeated over six planting ‘generations’. We selected *B*. *rapa* for this study, since it has been used in previous microbiome selection studies (Swenson *et al*., 2000; Panke‐Buisse *et al*., 2015) and there is significant interest in growing *Brassica* species in saline conditions (Pavlović *et al*., 2019). To build off previous studies of progressive microbiome selection (Swenson *et al*., 2000), we targeted high *B*. *rapa* biomass production as a phenotype, which is also a relevant agricultural trait. We used three selection treatments: four replicate phenotype‐selected lines, in which microbiomes were transferred forward from the roots of the highest biomass plants; a random selection control, in which microbiomes were transferred forward from plant roots from four randomly selected pots; and, uniquely, an add‐back control, in which the inoculated microbiome was always a thawed version of the initial soil used to initiate generation 1. We applied selection with and without salt amendment to alter the reliance of plants on soil microorganisms to mitigate stress. We hypothesized that patterns in microbiome selection would parallel those observed for plant biomass, with selection being more stable under salt disturbance.

## Materials and Methods

### Establishment of microbiome selection system

**Fig. 1:**
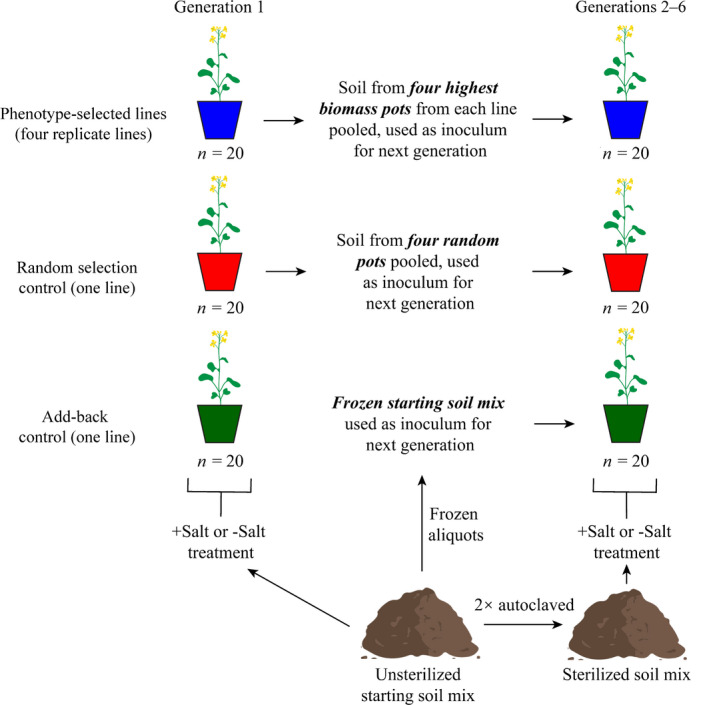
Concept figure of microbiome selection.

In October 2015, we collected fallow soil from Caldwell Farm on the Cornell University campus, as in Kaminsky *et al*. (2018). To our knowledge, the collected soil was not already under salt stress, but fertilizer and road salt accumulation on nearby grass could certainly have contributed to high salt pressure in some soil patches. Soil was dried and sieved to 4 mm and homogenized 9 : 8 (by volume) with Lambert LM‐111 All‐Purpose Mix (final mix: pH = 5.53; Total *n* = 0.2%; aluminum (Al) = 26.79 mg kg^−1^; calcium (Ca) = 2198.11 mg kg^−1^; potassium (K) = 192.56 mg kg^−1^; magnesium (Mg) = 320.07 mg kg^−1^; manganese (Mn) = 55.45 mg kg^−1^; P = 16.09 mg kg^−1^). Soil for planting generation 1 (G1) was used directly (Fig. 1), while the remainder was frozen at −15°C for later use. We produced > 30 000 *Brassica rapa* L. seeds in a single planting generation before the start of the experiment, and used this as a common seed pool for all experimental generations.

Soil for G1 was split into ‘no‐salt’ and ‘salt‐amended’ treatments. For salt‐amended soils, NaCl was added to achieve an electrical conductivity (EC) value of 4.1 mS cm^−1^ (the EC of no‐salt soils was 0.6 mS cm^−1^), which depressed *B*. *rapa* growth to *c*. 1/3 of that observed in no‐salt soils after 32 d. We chose to use a static pressure in order to reflect field conditions and to assess phenotypic change relative to a control over time. For both salt treatments, we filled 120 4‐inch (101.6 mm) diameter pots with 300 ml of soil, which had first been coated with vermiculite to limit water loss and microbial influx. Each pot was seeded with four evenly spaced *B*. *rapa* seeds that had been treated with 10% commercial bleach for 15 min, followed by five washes in sterile water.

Seeded pots were assigned randomly to one of six lines for each salt treatment (12 lines total with 20 pots each) and arranged randomly within 20 blocks in a walk‐in growth chamber at the Plant Growth Chamber Lab at Weill Hall (Cornell University, Ithaca, NY, USA). Within each salt treatment, these lines consisted of four replicate phenotype‐selected lines (i.e. microbiomes transferred based on high *B*. *rapa* biomass), a random selection control (i.e. randomly selected microbiomes transferred), and an add‐back control (i.e. initial soil thawed and then added as inoculum; Fig. 1). Plants were grown under a 30°C : 20°C, 16 h : 8 h, light : dark cycle, with light supplied at 335 μmol m^−2^ s^−1^ during the day and relative humidity set to 70%. Plants were grown for 32 d and watered daily with autoclaved deionized H_2_O. Based on mean daily water loss, no‐salt pots received 30 ml sterile water each day while salt‐amended pots received 15 ml sterile water each day. Water addition amounts were based on the amount of water needed to achieve soil saturation without leaching.

### Forward transfer of microbiomes

After 32 d, aboveground biomass was clipped at the soil, placed in paper bags, and dried at 75°C for 24 h. Pots were enclosed in individual air‐tight bags and stored at 4°C during the drying of plant material. After drying, aboveground biomass was weighed, and high biomass as well as random pots were retrieved for the forward transfer of microbiomes in the phenotype‐selected lines and random selection control lines, respectively.

To prepare potting substrate for planting generations 2–6 (G2–G6), the initial soil mix was thawed for 2 d, twice autoclaved for 1 h at 121°C with 24 h in between, and then inoculated with selected microbiomes from the previous generation. To transfer microbiomes, we mixed soil from the previous generation with the newly autoclaved soil at a ratio of 1 : 19. Although direct soil transfer will move both microorganisms and a small amount of the source soil to the recipient soil (Howard *et al*., 2017; Trexler & Bell, 2019), we aimed to approximate the approach used in previous studies (e.g. Lau & Lennon (2012)).

To generate inoculum for the phenotype‐selected lines, we homogenized equal volumes of root‐associated soil from four pots selected based on high *B. rapa* biomass production, excluding the 2 cm of soil at the top, sides, and bottom of each pot. Root‐associated soil was homogenized by manually agitating soil in a Ziploc bag. In order to limit any spatial biases (e.g. due to differences in humidity, temperature, etc.), we selected the top‐performing pot from each set of four blocks within each line, spatially across the bench. To generate inoculum for the random selection control, we used a random number generator to select a pot from within each set of four blocks. To generate inoculum for the add‐back control, we thawed the initial soil for 2 d before introducing it 1 : 19 to the autoclaved soil, as with the selected lines. At the end of each generation, soil was collected from the four high biomass pots for each line and the controls for DNA extraction and sequencing.

### DNA extraction, polymerase chain reaction (PCR) amplification, sequencing, and initial sequence processing

We extracted DNA from *c*. 0.25 g of homogenized rhizosphere soil using the MoBio PowerSoil DNA Isolation Kit (Qiagen). Downstream processing for sequencing and initial sequence analysis were as described in detail by Kaminsky *et al*. (2018). Sequencing was performed at the Cornell Genomics Center. We rarefied 16S and ITS data to 1155 and 775 sequences per sample, respectively. These thresholds were chosen to prevent the omission of any samples from the dataset, while normalizing read numbers per sample. The resulting dataset was then transformed compositionally and used to produce a phyloseq object (McMurdie & Holmes, 2013) for further analyses.

### Statistical analyses

To identify differences in plant biomass in particular generations, a Kruskal–Wallis test from the stats package was used (R Core Team, 2012). Alpha diversity comparisons were performed with a Kruskal–Wallis test for species richness (Chao1), diversity (Shannon diversity) and evenness (Pielou). To compare microbial composition between different generations and between the phenotype‐selected lines and controls, a principal coordinates analysis (PCoA) with a Bray–Curtis dissimilarity index was used. Ordinations were performed by the Phyloseq package (McMurdie & Holmes, 2013). Patterns elucidated by the PCoA were statistically compared using the ‘adonis’ (PERMANOVA) function in the vegan package with 999 permutations (Oksanen *et al*., 2015). To identify divergence of microbial composition from the add‐back control, a Kruskal–Wallis test was used with Bray–Curtis dissimilarities. To identify whether specific higher taxa were significantly different between the selected lines and the add‐back control for generations with significantly different plant biomass, a Kruskal–Wallis test was used. To examine forward transfer of microbial compositions between generations, Bray–Curtis dissimilarities were extracted for paired generations for each individual line (e.g. line 1 generation 2 vs line 1 generation 3). To examine operational taxonomic units (OTUs) that were either selected or filtered by the successive selection we performed differential abundance testing using deseq2 (Love *et al*., 2014). To identify taxa that were consistently associated with the phenotype‐selected lines, we performed network analysis at the genus level using the Phylosmith package (Smith, 2019). All statistical tests were performed in the R statistical environment (R Core Team, 2012).

## Results

### Plant performance did not consistently improve through time

**Fig. 2:**
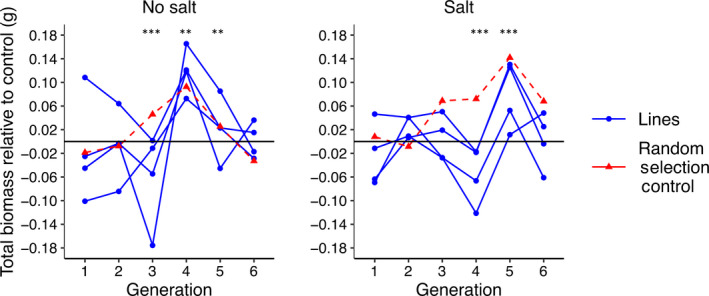
Biomass changes relative to the add‐back control in each generation. Asterisks indicate significant differences for the overall comparisons as follows: *, *P* < 0.05; **, *P* < 0.01; ***, *P* < 0.001. ‘Lines’ refers to phenotype‐selected lines. Each point represents the mean value of 20 pots.

The transfer of high biomass microbiomes through subsequent planting generations did not lead to a consistent improvement in plant biomass, at least within six planting generations. Generally, plant biomass outcomes appeared stochastic (Fig. 2; Supporting Information Figs S1, S2). Differences in plant biomass relative to the add‐back control were observed in G3, G4 and G5, and G4 and G5, in the no‐salt and salt‐amended treatments, respectively (Tables S1–S3). However, these biomass differences included both increases and decreases in biomass relative to the add‐back control.

### Selection drives reductions in taxonomic diversity

In general, differences observed in Shannon diversity (Fig. S3) were driven by differences in evenness (Fig. S4; Table S4). Overall, there was a decreasing trend in species diversity over planting generations except for bacterial composition in the no‐salt treatment. Notably, bacterial species diversity was lower in the salt‐amended treatment relative to the no‐salt treatment (Table S5). The add‐back control fungal species diversity was generally greater in G5 and G6 relative to all other groups (Tables S6, S7).

### Unlike plant biomass, microbial composition consistently diverges from the add‐back control across generations

**Fig. 3:**
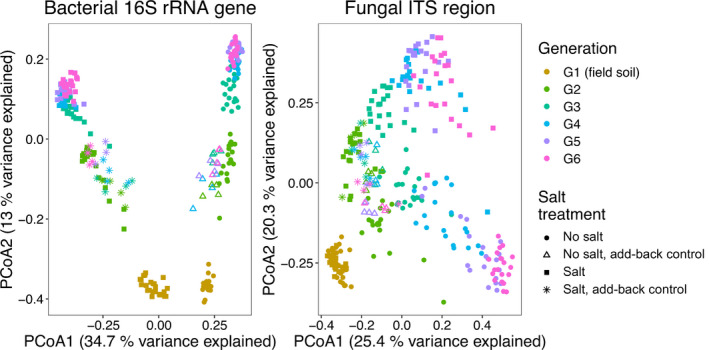
Principal coordinates analysis (PCoA) ordinations of bacterial (16S rRNA gene) and fungal (ITS region) composition over time and in the presence or absence of salt. Samples are colored by generation, and the shape of each symbol indicates the salt treatment. Add‐back control samples have unique shapes (hollow triangles for no‐salt and asterisks for salt‐amended) and cluster together (G2 onwards) despite the generational differences observed for the selected lines. G1 soil was derived from the field (including for the add‐back control).

Bacterial and fungal composition were significantly influenced by the presence or absence of salt, with ordinations identifying discrete clustering for each treatment (Fig. 3; Table S8). Generation was also significantly associated with composition (Table S8), with microbial composition diverging progressively from what was observed in G1 (Fig. 3). Salt explained comparatively more variance in bacterial composition (*R* ^2^ = 0.31), whereas differences across generations explained more of the variance for fungal composition (*R* ^2^ = 0.29).

When microbial composition was separated by generation, microbial composition significantly differed overall in all generations except G1 (Table S9). As G1 soil was derived from the field we expected no microbial composition differences, but we did observe a difference for bacterial composition in the no‐salt treatment. Differences between the random selection control and the phenotype‐selected lines were primarily observed for bacterial composition rather than fungal composition. By contrast, microbial composition differences between the add‐back control and the individual phenotype‐selected lines were consistently observed (Tables S10, S11). Comparisons between the add‐back control and phenotype‐selected lines explained more variance in microbial composition (*R* ^2^ average in G6 = 0.60) relative to comparisons involving the random selection control and phenotype‐selected lines (*R* ^2^ average in G6 = 0.34), possibly indicating microbial composition divergence from the add‐back control.

**Fig. 4:**
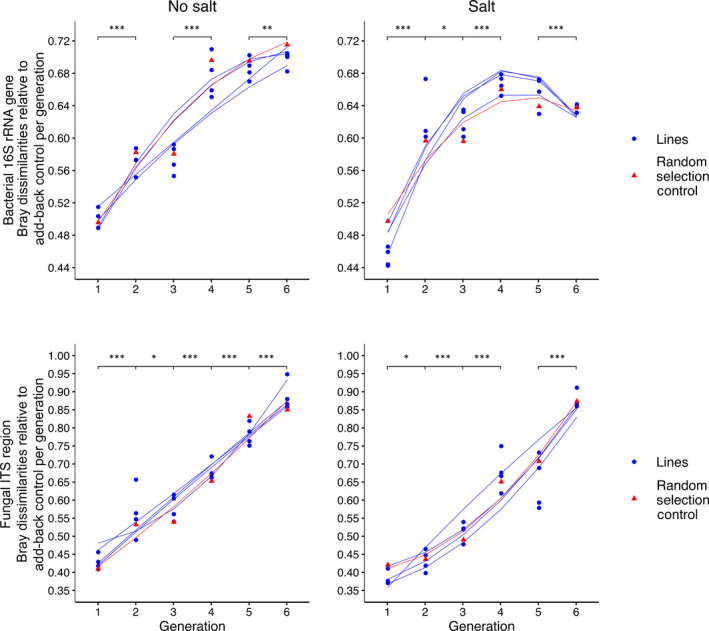
Divergence of microbial composition relative to the add‐back control. Bray–Curtis dissimilarities between the add‐back control and selected lines were extracted for each generation. Data was plotted with a polynomial line. As soil from G1 was derived from the field, we would not expect dissimilarity to differ for this generation. Asterisks indicate significant differences (*, *P* < 0.05; **, *P* < 0.01; ***, *P* < 0.001).

To explore possible divergence of microbial composition from the add‐back control, we extracted Bray–Curtis dissimilarities of the add‐back control vs all selected lines within each generation (Fig. 4). Microbial composition diverged increasingly from the add‐back control across generations (Table S12). By G6, fungal composition was more dissimilar from the corresponding add‐back control than was bacterial composition (Fig. 4). Dissimilarity in fungal composition trended upwards through time, whereas dissimilarity in bacterial composition initially trended upwards and then appeared to plateau (no‐salt) or decline slightly (salt‐amended).

To determine which higher taxa were driving the divergence between the add‐back control and the phenotype‐selected lines and random selection control, we examined the linear trajectories of higher taxa (Figs S5–S7). Generally, the relative abundance of bacterial phyla begins to converge over time and may explain the plateau and decline of Bray–Curtis dissimilarity in the bacterial composition data. In the no‐salt treatment, the relative abundance of an unclassified class in the Ascomycota continually diverged from the add‐back control, mirroring the Bray–Curtis dissimilarity fungal composition divergence patterns (Fig. 4). In the salt‐amended treatment, divergence could be explained by the Saccharomycetes in the earlier generations and the Sordariomycetes in the later generations. We performed differential abundance testing to identify OTUs that were selected or filtered by the phenotype‐selected lines (Tables S13–S15) by comparing the phenotype‐selected lines to the add‐back control. Irrespective of salt amendment, we identified 32 bacterial OTUs and 10 fungal OTUs that were significantly positively selected by successive selection with the phenotype‐selected lines. By comparison, 17 bacterial OTUs and 17 fungal OTUs were significantly filtered out during the successive selection. The salt‐amended treatment had a greater number of positively selected microorganisms, with 19 bacterial OTUs and 7 fungal OTUs, compared to 13 bacterial OTUs and 3 fungal OTUs being selected in the no‐salt treatment. We observed no significantly different OTUs between the phenotype‐selected lines and the random selection control. Additionally, we identified six genera that were consistently significantly associated with the grouped phenotype‐selected lines using network analysis (Table S16). Interestingly, correlations during generations 4 and 5 appeared to invert for each taxon, suggesting that plant filtering may be stronger during these generations.

## Discussion

Climate‐driven salinization of soils is expected to exacerbate existing salinization issues within intensively managed lands and under natural conditions (Eswar *et al*., 2021). In agricultural settings, there is significant interest in enhancing our ability to grow crops, like *Brassica* species, in salt‐heavy soils (Pavlović *et al*., 2019) to adapt to increasing soil salinization. Microbial contributions to salt tolerance are likely driven by the additive contributions of many taxa within the microbiome (Kumar *et al*., 2020). Approaches to select for salt‐tolerance functions likely require an approach that simultaneously targets many taxa and their interactions, making the manipulation of microbiomes, with the goal of altering microbiome function, an appealing concept in agriculture (Parnell *et al*., 2016; Bell *et al*., 2019; Kaminsky *et al*., 2019). Many recent studies link microbial composition to plant phenotypic traits (Lau & Lennon, 2012; Wagner *et al*., 2014; Panke‐Buisse *et al*., 2015), suggesting that artificial selection of microbiomes may be able to alter plant phenotypes in a predictable manner. Such an approach has previously been shown to influence plant biomass (Swenson *et al*., 2000; Kaminsky *et al*., 2018), resilience to drought stress (Lau & Lennon, 2012), and plant flowering time (Lau & Lennon, 2012; Panke‐Buisse *et al*., 2015). However, it has not been clear whether artificial selection for microbiome traits is stable over time or whether the trajectory of microbial composition parallels changes in plant phenotypic traits. Sporadic inter‐generational changes in plant phenotype have been reported when artificially selecting for soil microbiomes (Swenson *et al*., 2000; Panke‐Buisse *et al*., 2015).

We observed consistent and near‐linear divergence of microbial composition from the add‐back control in both phenotype‐selected lines and the random selection control. The divergence of microbial composition is likely driven by the successive selection of positive, neutral, and deleterious microorganisms that preferentially colonize the plant root and rhizosphere (Shi *et al*., 2015; Tkacz *et al*., 2015; Zhalnina *et al*., 2018; King *et al*., 2021), as opposed to differential selection based on observed plant phenotypes. The lack of significance for any OTUs between the random selection control and phenotype‐selected lines (i.e. differential abundance testing) further highlights that we were likely accumulating rhizosphere‐adapted microorganisms. Selection of bacteria under added salt appeared to plateau more quickly than in the no‐salt treatment (Fig. 4), suggesting that plant selection of available microbes occurred more quickly under saline conditions. In agreement with this conclusion, differential abundance testing also identified greater numbers of OTUs that were positively selected under the salt‐amendment treatment. Salt stress also explained more of the beta‐diversity variance in bacterial composition relative to fungal composition, suggesting that soil salinization plays a greater role in bacterial filtering. In our experimental design, we suspect that there are two stages of microbiome selection which occur sequentially: continual filtering for rhizosphere‐adapted microorganisms, and periodic positive selection towards a particular phenotype. The more rapid plateau trend for bacterial selection under saline conditions (Fig. 4) suggests that the first selection stage had mostly been completed through six generations, and that perhaps a longer selection course would have identified more obvious phenotype selection.

Interestingly, although soil microbiome selection had variable impacts on plant performance, we observed no clear positive trend between plant biomass and either random or targeted microbiome selection under saline or no‐salt conditions, although plant biomass was higher than in our add‐back control in certain generations. Previous studies indicate that selecting for microbiomes based on opposing phenotypes (e.g. early vs late flowering) or selection conditions (e.g. low or high water availability) can yield microbiomes that impact plant growth differently (Swenson *et al*., 2000; Lau & Lennon, 2012; Wagner *et al*., 2014; Panke‐Buisse *et al*., 2015; Kaminsky *et al*., 2018). We show that, at least in the short‐term, there is no obvious difference between selection of microbiomes associated with high‐performing plants and selection of microbiomes associated with randomly selected pots. Due to our comprehensive microbiome sampling, we focused only on the first six generations of selection, but positive selection of plant traits through microbiome selection has been observed in as little as three to five generations (Swenson *et al*., 2000; Lau & Lennon, 2012; Panke‐Buisse *et al*., 2015). Although we hypothesized that phenotype selection would be more consistent in the salt‐amended treatment, due to increased selection pressure on the plants, biomass improvements were equally inconsistent across both salt treatments. One consideration for future microbiome‐selection studies is the choice of initial soil inoculum. In our study, we used fallow soil that was not under obvious salt pressure as a neutral inoculum source, to avoid biasing microbial selection in the salt‐amended treatment. Our goal was for the inoculum to act as a diverse microbial ‘seedbank’, without a substantial influence from field planting legacy. However, we acknowledge that inoculum source could certainly be important for the initial recruitment of beneficial taxa/functions during selection and would be an important target for future study.

In contrast to previous studies, we make use of four replicated phenotype‐selected lines, a random selection control, and an add‐back control. The use of an add‐back control provides a robust reference point for microbiome composition and plant phenotypic change across generations, which exceeds previous standards for selection experiments that involve multiple plant growth cycles. Even within highly controlled growth chambers, factors such as humidity and gas concentration can have a considerable impact on mean plant growth (Porter *et al*., 2015). Distinct clustering of add‐back control microbiomes across generations, in which the initial microbiome was used to inoculate sterilized soil (G2 onwards), suggests that freezing and re‐inoculating soil is an effective approach for reproducing soil microbiome composition, even 8 months after freezing (Fig. 3). In support of our observation, reestablishment of microbiome composition from frozen inocula has also been shown for the lettuce phyllosphere (Williams & Marco, 2014).

### Conclusion

Interest in microbial management and microbiome manipulation, for the purpose of improving plant fitness, has steadily increased in recent years. Our data demonstrate that selecting for plant phenotype through microbiome transfer can be unpredictable in the early stages of selection. Despite the unpredictable trajectory of plant phenotypes, we observed a near‐linear divergence in microbial composition relative to our add‐back control, likely driven by the accumulation of rhizosphere‐adapted microorganisms. This rapid divergence in microbial composition suggests that inadvertent microbial selection could occur quickly in agricultural settings, particularly in fields that repeatedly grow similar genotypes. Microbial composition in the rhizosphere is often unique across dissimilar plant types (Berg & Smalla, 2009), so the accumulation of rhizosphere‐adapted microorganisms for one particular crop has implications for both microbial management and crop rotations. Examinations of microbial recruitment by plants under saline conditions are vital for understanding how plant–microbial associations will be affected by global climate change. We observed stronger plant filtering for bacterial, as opposed to fungal, compositions under saline conditions. Our data indicate that salinization may impact the bacterial pools available for plant recruitment and may further exacerbate the decline in microbial function often observed in agricultural settings.

## Author contributions

THB conceived of and designed the study, with important input from JK‐K. LMK, MG, GLT and THB set up and managed the experiment and also collected and processed samples. WLK and THB analyzed the data. WLK and THB wrote the initial manuscript draft. All authors contributed to editing and approved the final manuscript.

## Supporting information


**Fig. S1** Biomass changes between phenotype‐selected lines, the random selection control and the add‐back control.
**Fig. S2** Flowering time and C : N ratio changes between the phenotype‐selected lines (Lines), the random selection control and the add‐back control.
**Fig. S3** Shannon diversity for bacterial and fungal operational taxonomic units OTUs.
**Fig. S4** Alpha diversity measures for bacterial and fungal composition data.
**Fig. S5** Relative abundance linear trajectories of the most abundant bacterial phyla and fungal classes throughout the generations.
**Fig. S6** Relative abundance linear trajectories for the three most abundant Proteobacterial classes and the Acidobacteria and Actinobacteria phyla throughout the generations.
**Fig. S7** Beta‐diversity plots for fungal and bacterial compositions.
**Table S1** Overall plant phenotypic data comparisons.
**Table S2** Plant biomass comparisons per generation for the no‐salt treatment.
**Table S3** Plant biomass comparisons per generation for the salt‐amended treatment.
**Table S4** Overall alpha diversity comparisons for species diversity (Shannon diversity), species evenness (Pielou) and species richness (Chao1).
**Table S5** 16S rRNA alpha diversity comparisons for the significantly different generations in the salt‐amended treatment.
**Table S6** ITS alpha diversity comparisons for the significantly different generations in the no‐salt treatment.
**Table S7** ITS alpha diversity comparisons for the significantly different generations in the salt‐amended treatment.
**Table S8** Overall beta‐diversity comparisons for bacterial and fungal composition at the OTU level based on PERMANOVA.
**Table S9** Overall beta diversity comparisons for individual generations.
**Table S10** Bacterial composition beta diversity comparisons.
**Table S11** Fungal composition beta diversity comparisons.
**Table S12** Comparison of Bray–Curtis dissimilarities relative to the add‐back control between generations.
**Table S13** Bacteria that were successively selected by the phenotype‐selected lines.
**Table S14** Bacteria that were successively filtered by the phenotype‐selected lines.
**Table S15** Fungi that were successively selected by the phenotype‐selected lines.
**Table S16** Microbial genera that were consistently selected or filtered over time by the phenotype‐selected lines.
Please note: Wiley Blackwell are not responsible for the content or functionality of any Supporting Information supplied by the authors. Any queries (other than missing material) should be directed to the *New Phytologist* Central Office.

## Data Availability

MiSeq data have been stored in the NCBI Sequence Read Archive and can be found under project no. PRJNA658825.
