# Supplementary material for: Soil salinization accelerates microbiome stabilization in iterative selections for plant performance
Source: New Phytol. 2021 Oct 26;234(6):2101–10. doi: 10.1111/nph.17774 (PMC9297847; doi:10.1111/nph.17774)
Supplement: Supplementary file 1 — Fig. S1 Biomass changes between phenotype‐selected lines, the random selection control and the add‐back control. Fig. S2 Flowering time and C : N ratio changes between the phenotype‐selected lines (Lines), the random selection control and the add‐back control. Fig. S3 Shannon diversity for bacterial and fungal operational taxonomic units OTUs. Fig. S4 Alpha diversity measures for bacterial and fungal composition data. Fig. S5 Relative abundance linear trajectories of the most abundant bacterial phyla and fungal classes throughout the generations. Fig. S6 Relative abundance linear trajectories for the three most abundant Proteobacterial classes and the Acidobacteria and Actinobacteria phyla throughout the generations. Fig. S7 Beta‐diversity plots for fungal and bacterial compositions. Table S1 Overall plant phenotypic data comparisons. Table S2 Plant biomass comparisons per generation for the no‐salt treatment. Table S3 Plant biomass comparisons per generation for the salt‐amended treatment. Table S4 Overall alpha diversity comparisons for species diversity (Shannon diversity), species evenness (Pielou) and species richness (Chao1). Table S5 16S rRNA alpha diversity comparisons for the significantly different generations in the salt‐amended treatment. Table S6 ITS alpha diversity comparisons for the significantly different generations in the no‐salt treatment. Table S7 ITS alpha diversity comparisons for the significantly different generations in the salt‐amended treatment. Table S8 Overall beta‐diversity comparisons for bacterial and fungal composition at the OTU level based on PERMANOVA. Table S9 Overall beta diversity comparisons for individual generations. Table S10 Bacterial composition beta diversity comparisons. Table S11 Fungal composition beta diversity comparisons. Table S12 Comparison of Bray–Curtis dissimilarities relative to the add‐back control between generations. Table S13 Bacteria that were successively selected by the phenotype‐selected lines. Table [file NPH-234-2101-s001.pdf]

***New Phytologist* Supporting Information**

Article title: **Soil salinization accelerates microbiome stabilization in iterative selections for plant performance**

Authors: William L. King, Laura M. Kaminsky, Maria Gannett, Grant L. Thompson, Jenny Kao-Kniffin, Terrence H. Bell

Article acceptance date: 29 September 2021

The following Supporting Information is available for this article:

Fig. S1 Biomass changes between Phenotype-Selected Lines, the Random Selection Control and the Add-back Control.

Fig. S2 Flowering time and C:N ratio changes between the Phenotype-Selected Lines (Lines), the Random Selection Control and the Add-back Control.

Fig. S3 Shannon diversity for bacterial and fungal OTUs.

Fig. S4 Alpha diversity measures for bacterial and fungal composition data.

Fig. S5 Relative abundance linear trajectories of the most abundant bacterial Phyla and fungal Classes throughout the Generations.

Fig. S6 Relative abundance linear trajectories for the three most abundant Proteobacterial classes and the Acidobacteria and Actinobacteria phyla throughout the Generations.

Fig. S7 Beta-diversity plots for fungal and bacterial compositions.

Table S1 Overall plant phenotypic data comparisons.

Table S2 Plant biomass comparisons per generation for the No Salt treatment.

Table S3 Plant biomass comparisons per generation for the Salt treatment.

Table S4 Overall alpha diversity comparisons for Species Diversity (Shannon diversity), Species Evenness (Pielou) and Species Richness (Chao1).

Table S5 16S rRNA alpha diversity comparisons for the significantly different Generations in the Salt treatment.

Table S6 ITS alpha diversity comparisons for the significantly different Generations in the No Salt treatment.

Table S7 ITS alpha diversity comparisons for the significantly different Generations in the Salt treatment.

Table S8 Overall beta-diversity comparisons for bacterial and fungal composition at the OTU level based on PERMANOVA.

Table S9 Overall beta diversity comparisons for individual generations.

Table S10 Bacterial composition beta diversity comparisons.

Table S11 Fungal composition beta diversity comparisons.

Table S12 Comparison of Bray Curtis dissimilarities relative to the Add-back Control between Generations.

Table S13 Bacteria that were successively selected by the Phenotype-Selected Lines.

Table S14 Bacteria that were successively filtered by the Phenotype-Selected Lines.

Table S15 Fungi that were successively selected by the Phenotype-Selected Lines.

Table S16 Microbial genera that were consistently selected or filtered over time by the Phenotype-Selected Lines.

Fig. S1 Biomass changes between Phenotype-Selected Lines, the Random Selection Control and the Add-back Control. Pod and Stem biomass were only collected from Generation 3. As the inputs from one Generation depended on biomass data from the previous Generation, we had thought time would be limited to collect pod and stem biomass. However, we discovered it was feasible and began collection at Generation 3 onwards. Note the difference in y-axis scales for the No-salt and corresponding Salt treatment panels.

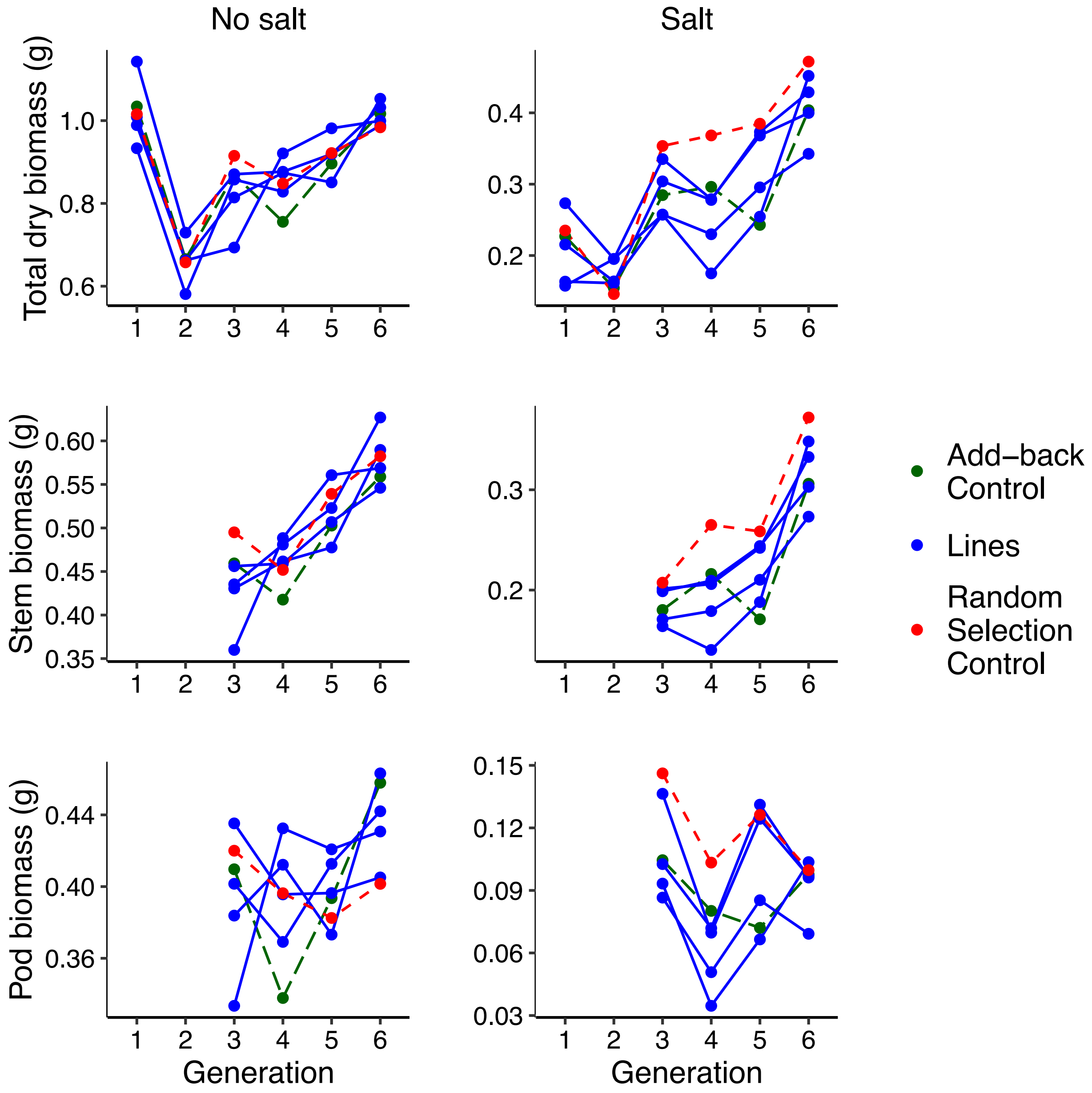

Fig. S2 Flowering time and C:N ratio changes between the Phenotype-Selected Lines (Lines), the Random Selection Control and the Add-back Control. Pod and stem C:N ratios were only collected in Generation 5. Note the difference in y-axis scales for the No-Salt and corresponding Salt treatment panels.

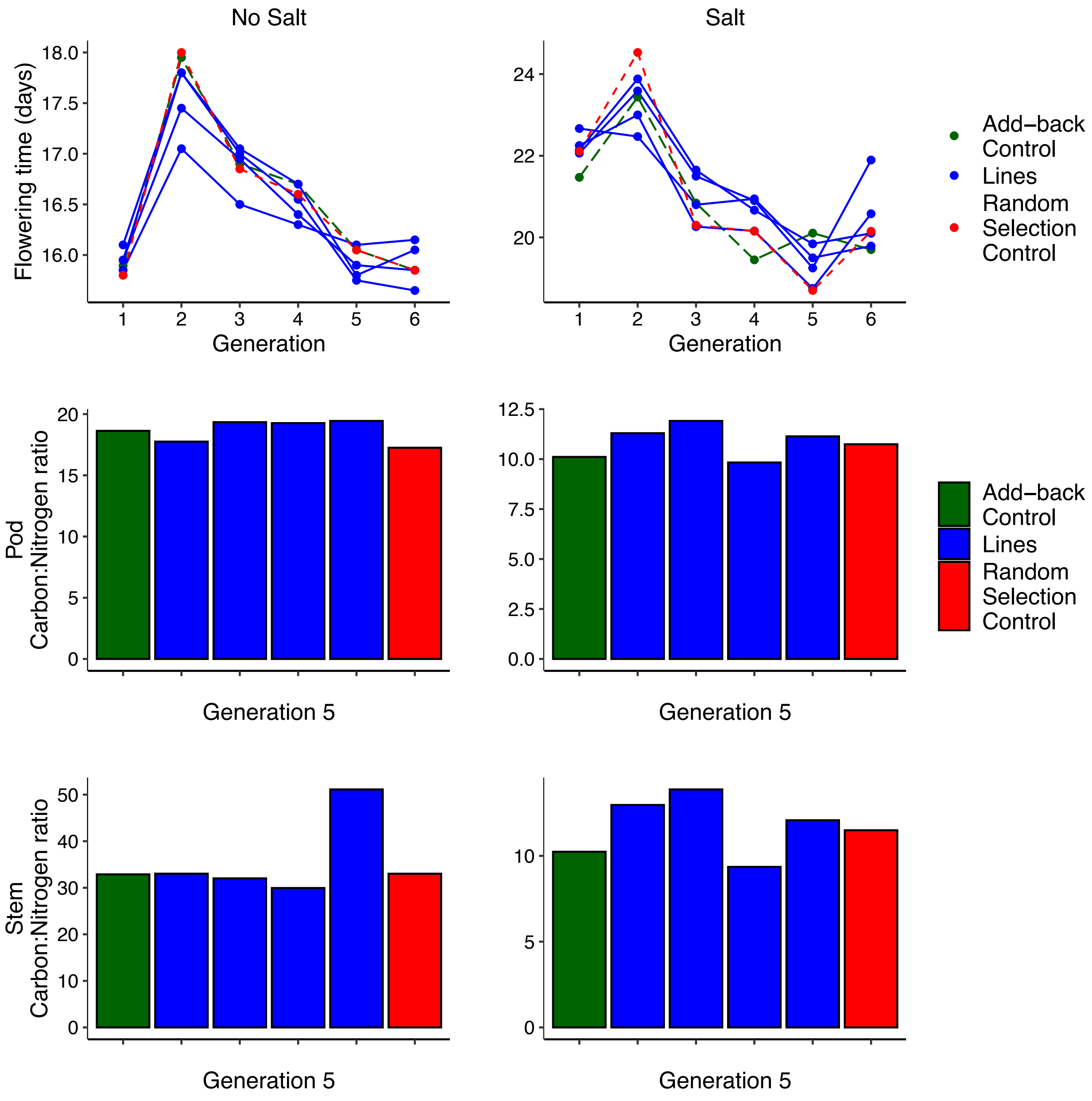

Fig. S3 Shannon diversity for bacterial and fungal OTUs. Overall comparison p-values: ** = <0.01, * = <0.05. Lines refers to Phenotype-Selected Lines. Shown data is mean ± standard error.

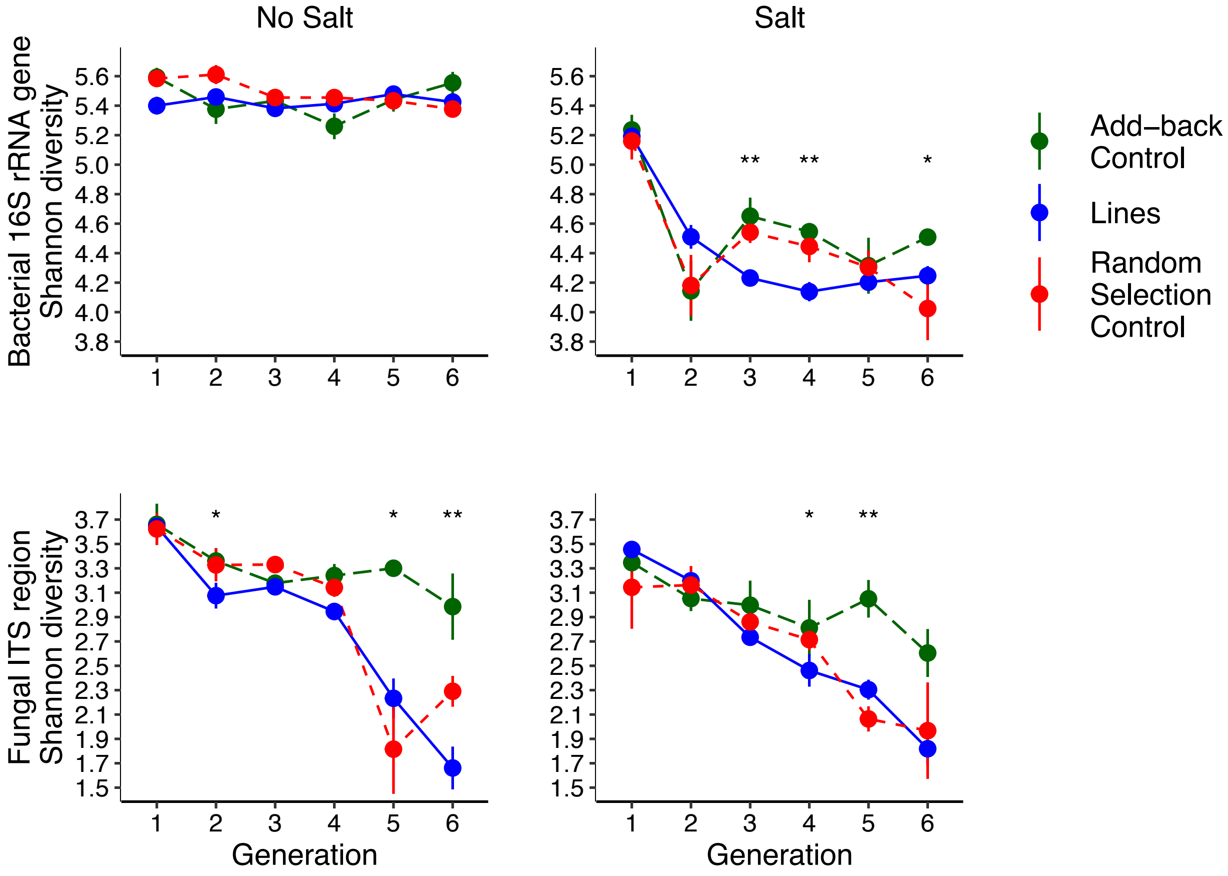

Fig. S4 Alpha diversity measures for bacterial and fungal composition data.

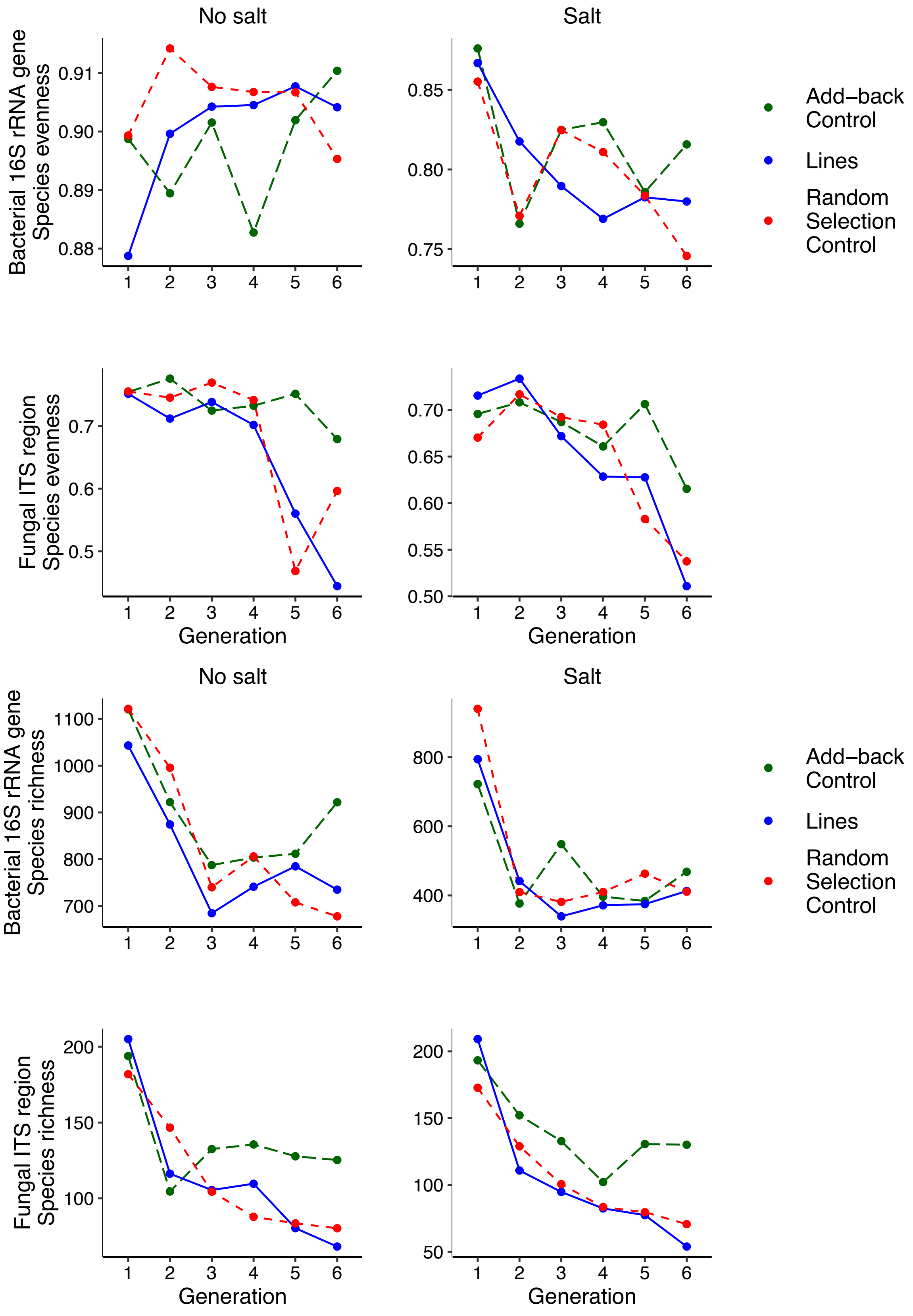

Fig. S5 : Relative abundance linear trajectories of the most abundant bacterial Phyla and fungal Classes throughout the Generations. Generations 2 to 6 are shown. The four most abundant taxa in each Salt treatment were chosen. Shown data is mean ± standard error. Lines refers to the averaged Phenotype-Selected Lines.

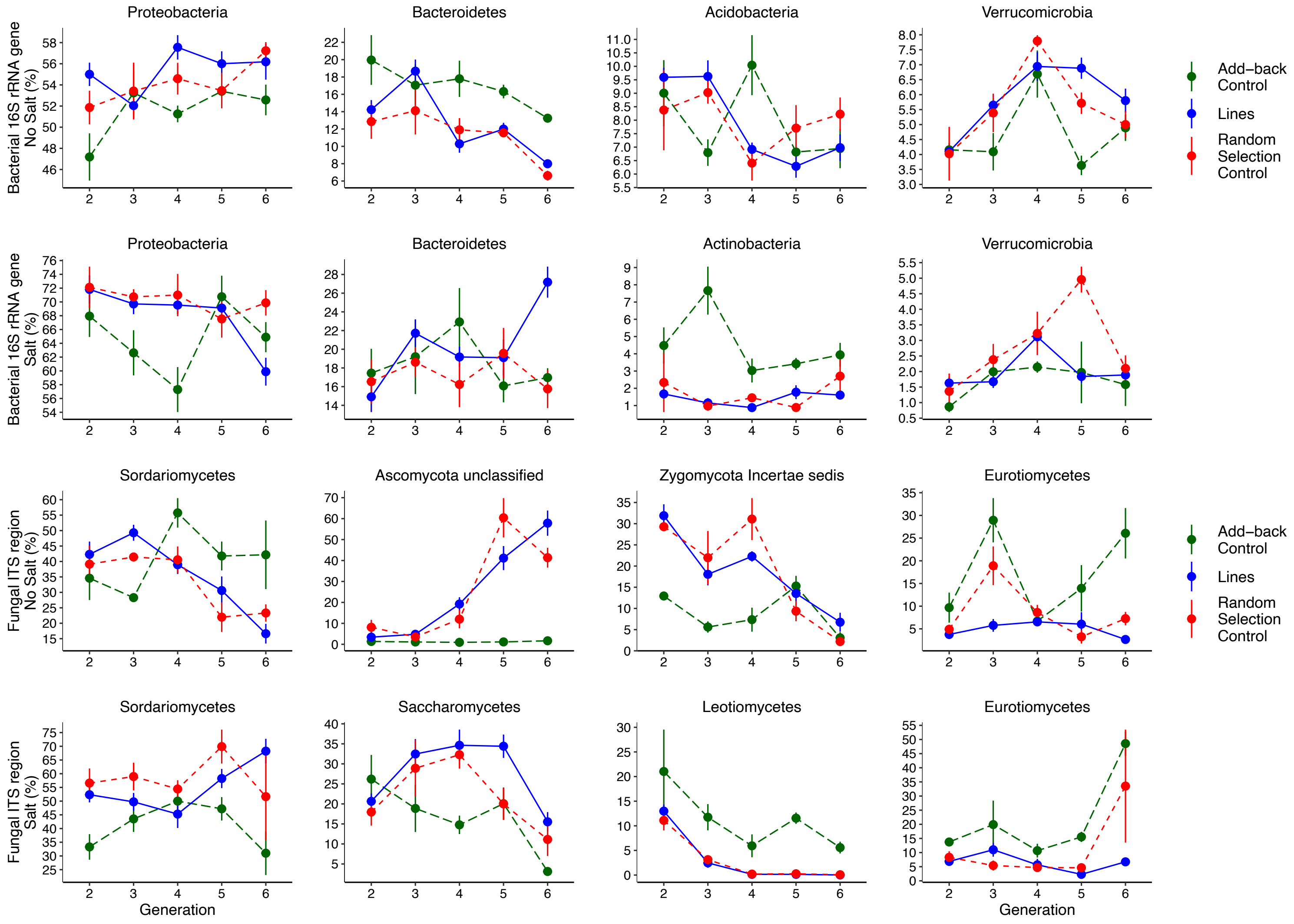

Fig. S6 Relative abundance linear trajectories for the three most abundant Proteobacterial classes and the Acidobacteria and Actinobacteria phyla throughout the Generations. Generations 2 to 6 are shown. Shown data is mean ± standard error. Lines refers to the averaged Phenotype-Selected Lines.

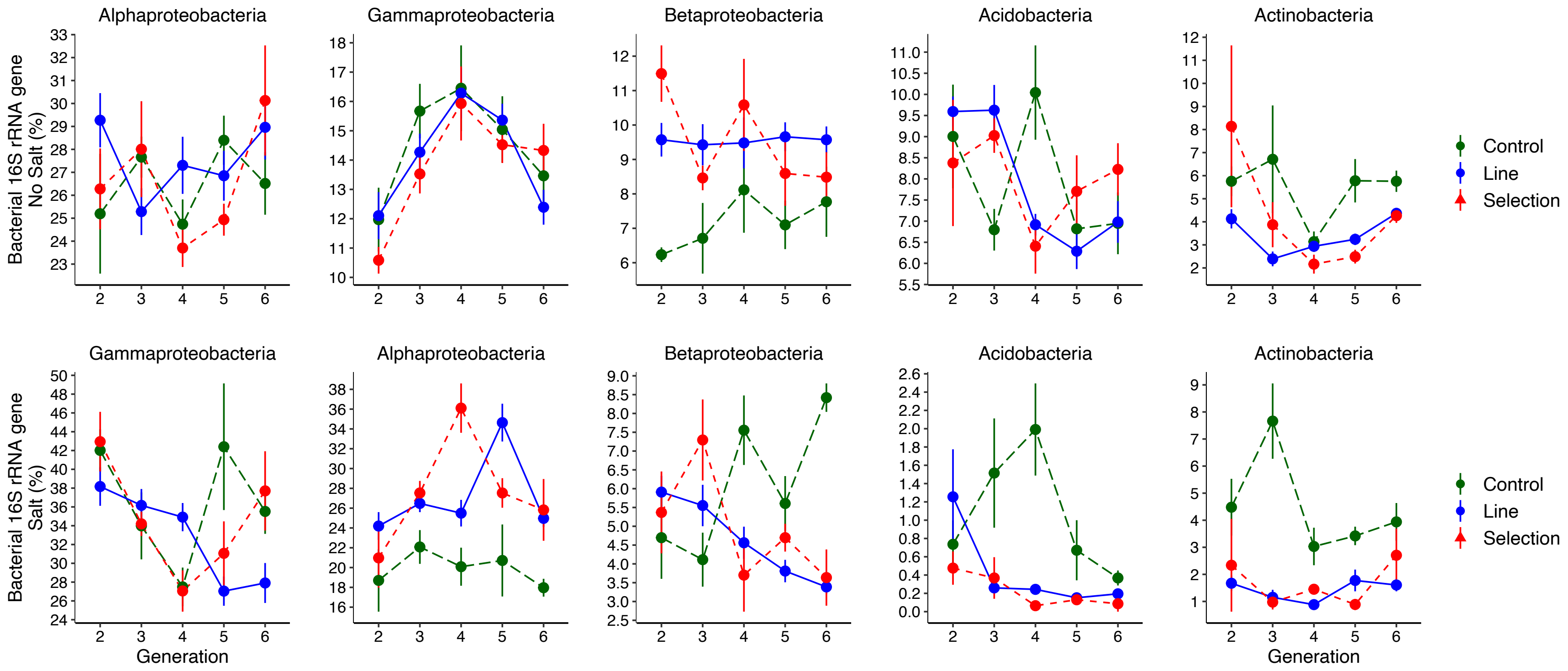

Fig. S7 Beta-diversity plots for fungal and bacterial compositions. Data were summarized at the Class level for fungal data (A) and the Phylum level for bacterial data (B). The ten most abundant taxa are shown. X-axis is ordered by Generation 1 to 6.

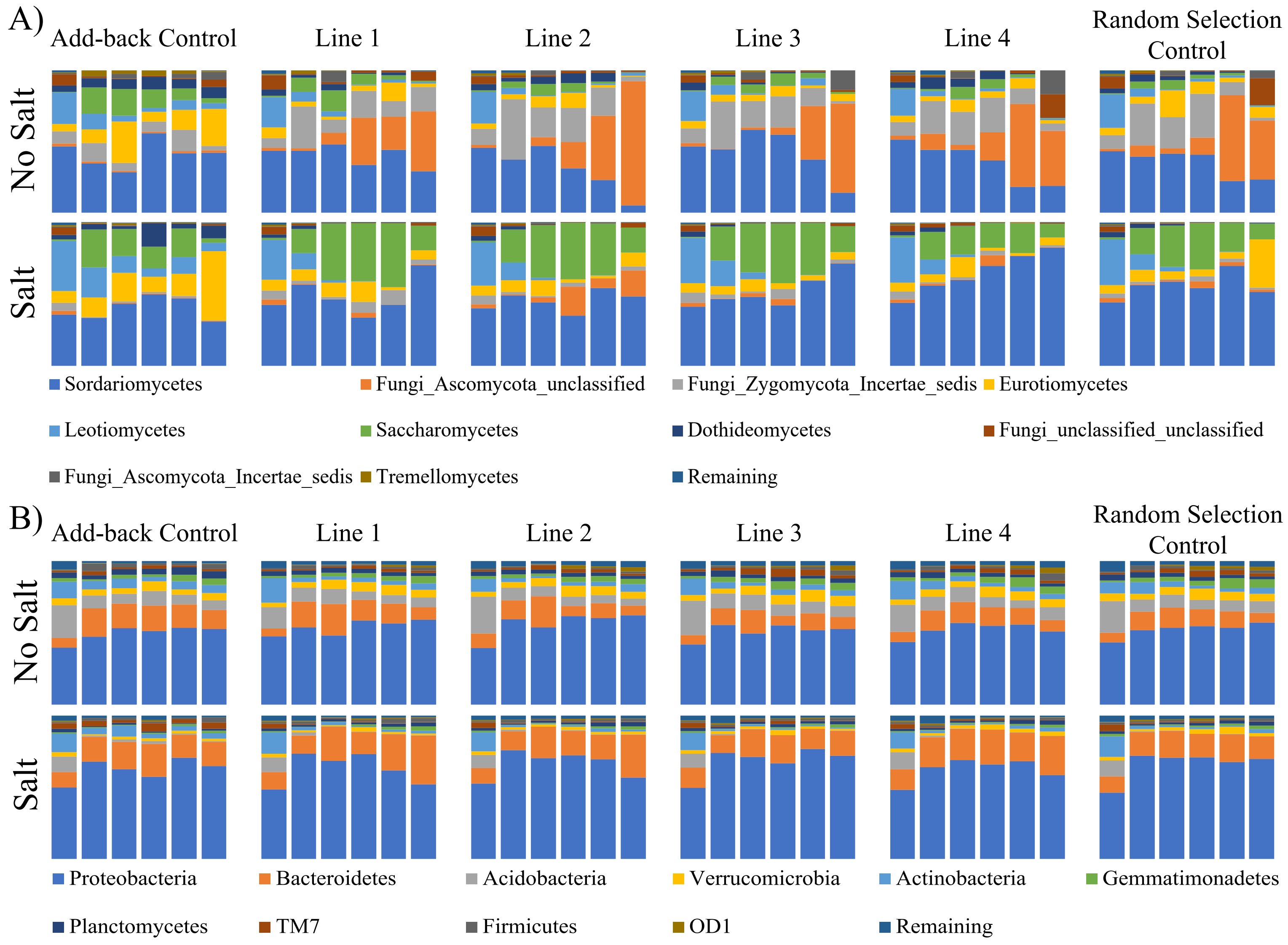

Table S1 Overall plant phenotypic data comparisons. All comparisons are 5 degrees of freedom. NS is no significance. C:N ratio was only collected in Generation 5. NA is not applicable as no overall significance was identified.

No Salt	Generation 1	Generation 2	Generation 3	Generation 4	Generation 5	Generation 6
Total biomass	NS	NS	$H = 23$, $p < 0.001$	$H = 19$, $p = 0.002$	$H = 18$, $p = 0.002$	NS
Stem biomass	NS	NS	$H = 28$, $p < 0.001$	NS	$H = 13$, $p = 0.03$	NS
Pod biomass	NS	NS	$H = 12$, $p = 0.04$	$H = 13$, $p = 0.02$	NS	NS
Flowering time	NS	NS	NS	NS	NS	$H = 13$, $p = 0.03$
Pod C:N ratio	NA	NA	NA	NA	NS	NA
Stem C:N ratio	NA	NA	NA	NA	NS	NA
Salt	Generation 1	Generation 2	Generation 3	Generation 4	Generation 5	Generation 6
Total biomass	NS	NS	NS	$H = 21$, $p < 0.001$	$H = 21$, $p < 0.001$	NS
Stem biomass	NS	NS	NS	$H = 18$, $p = 0.003$	$H = 13$, $p = 0.02$	NS
Pod biomass	NS	NS	NS	$H = 22$, $p < 0.001$	$H = 25$, $p < 0.001$	NS
Flowering time	NS	NS	$H = 15$, $p = 0.009$	NS ($p = 0.06$)	$H = 12$, $p = 0.03$	$H = 18$, $p = 0.003$
Pod C:N ratio	NA	NA	NA	NA	NS	NA
Stem C:N ratio	NA	NA	NA	NA	NS	NA

Table S2 Plant biomass comparisons per generation for the No Salt treatment. All comparisons are 1 degree of freedom. NS is no significance and NA is not applicable as no overall significance was identified. Data is shown as p-value (H-value). Only those Generations with a significant overall difference between all groups are shown. Only comparisons relative to selection and the add-back control are shown.

No Salt	Group	Lines	Generation 3	Generation 4	Generation 5
Total biomass	Random Selection Control	Line1	<0.001 (17)	0.03 (5)	NS
		Line2	0.05 (4)	NS	NS
		Line3	NS	NS	NS
		Line4	NS	NS	NS
		Add-back Control	NS	0.02 (5)	NS
	Add-back Control	Line1	0.001 (11)	<0.001 (17)	0.002 (10)
		Line2	NS	0.009 (7)	0.05 (4)
		Line3	NS	0.003 (9)	NS
		Line4	NS	0.05 (4)	NS
		Random Selection	NS	0.02 (5)	NS
No Salt	Group	Lines	Generation 3	Generation 4	Generation 5
Stem biomass	Random Selection Control	Line1	<0.001 (22)	NA	NS
		Line2	0.01 (6)	NA	NS
		Line3	0.003 (9)	NA	NS
		Line4	0.03 (5)	NA	NS
		Add-back Control	NS	NA	NS
	Add-back Control	Line1	<0.001 (13)	NA	0.008 (7)
		Line2	NS	NA	NS
		Line3	NS	NA	NS
		Line4	NS	NA	NS
		Random Selection	NS	NA	NS
No Salt	Group	Lines	Generation 3	Generation 4	Generation 5
Pod biomass	Random Selection Control	Line1	0.005 (8)	0.04 (4)	NA
		Line2	NS	NS	NA
		Line3	NS	NS	NA
		Line4	NS	NS	NA
		Add-back Control	NS	0.02 (5)	NA
	Add-back Control	Line1	0.02 (5)	0.002 (10)	NA
		Line2	NS	0.03 (5)	NA
		Line3	NS	0.04 (4)	NA
		Line4	NS	NS	NA
		Random Selection	NS	0.02 (5)	NA

Table S3 Plant biomass comparisons per generation for the Salt treatment. All comparisons are 1 degree of freedom. NS is no significance. Data is shown as p-value (H-value). Only those Generations with a significant overall difference between all groups are shown. Only comparisons relative to selection and the add-back control are shown.

Salt	Group	Lines	Generation 4	Generation 5
Total biomass	Random Selection Control	Line1	0.007 (7)	0.04 (4)
		Line2	<0.001 (15)	0.008 (7)
		Line3	0.04 (4)	NS
		Line4	0.04 (4)	NS
		Add-back Control	0.05 (4)	0.002 (10)
	Add-back Control	Line1	NS	NS
		Line2	0.002 (10)	NS
		Line3	NS	<0.001 (13)
		Line4	NS	0.005 (8)
		Random Selection	0.05 (4)	0.002 (10)
Salt	Group	Lines	Generation 4	Generation 5
Stem biomass	Random Selection Control	Line1	0.02 (5)	NS
		Line2	<0.001 (14)	0.05 (4)
		Line3	NS	NS
		Line4	NS	NS
		Add-back Control	NS	0.006 (7)
	Add-back Control	Line1	NS	NS
		Line2	0.003 (9)	NS
		Line3	NS	0.01 (7)
		Line4	NS	0.02 (6)
		Random Selection	NS	0.006 (7)
Salt	Group	Lines	Generation 4	Generation 5
Pod biomass	Random Selection Control	Line1	0.002 (9)	0.02 (6)
		Line2	<0.001 (15)	<0.001 (12)
		Line3	NS	NS
		Line4	0.05 (4)	NS
		Add-back Control	NS	0.002 (10)
	Add-back Control	Line1	0.02 (5)	NS
		Line2	0.001 (10)	NS
		Line3	NS	0.002 (10)
		Line4	NS	0.01 (7)
		Random Selection	NS	0.002 (10)

Table S4 Overall alpha diversity comparisons for Species Diversity (Shannon diversity), Species Evenness (Pielou) and Species Richness (Chao1). All comparisons were 5 degrees of freedom.

Gene	No Salt	Generation 1	Generation 2	Generation 3	Generation 4	Generation 5	Generation 6
16S	Diversity	NS	NS	NS	NS	NS	NS
	Evenness	NS	NS	NS	NS	NS	NS
	Richness	NS	NS	NS	NS	NS	NS
ITS	Diversity	NS	H = 15, p = 0.01	NS	NS	H = 13, p = 0.03	H = 15, p = 0.008
	Evenness	NS	H = 14, p = 0.02	NS	NS	H = 13, p = 0.02	H = 13, p = 0.02
	Richness	NS	NS	NS	H = 13, p = 0.02	NS	NS
**Gene**	**Salt**	**Generation 1**	**Generation 2**	**Generation 3**	**Generation 4**	**Generation 5**	**Generation 6**
16S	Diversity	NS	NS	H = 19, p = 0.002	H = 16, p = 0.008	NS	H = 13, p = 0.02
	Evenness	NS	NS	H = 14, p = 0.02	H = 14, p = 0.02	NS	H = 14, p = 0.01
	Richness	NS	NS	H = 15, p = 0.01	NS	NS	NS
ITS	Diversity	NS	NS	NS	H = 12, p = 0.03	H = 20, p = 0.001	NS
	Evenness	NS	NS	NS	H = 12, p = 0.03	H = 14, p = 0.02	NS
	Richness	NS	NS	NS	NS	H = 15, p = 0.01	H = 13, p = 0.03

Table S5 16S rRNA alpha diversity comparisons for the significantly different Generations in the Salt treatment. All comparisons are 1 degree of freedom. NS is no significance and NA is not applicable as no overall significance was identified. Data is shown as p-value (H-value). Only those Generations with a significant overall difference between all groups are shown. Only comparisons relative to selection and the add-back control are shown.

Salt	Group	Lines	Generation 3	Generation 4	Generation 6
16S Species diversity	Random Selection Control	Line1	0.02 (5)	NS	NS
		Line2	0.02 (5)	0.02 (5)	NS
		Line3	NS	NS	NS
		Line4	NS	NS	NS
		Add-back Control	NS	NS	NS
	Add-back Control	Line1	0.02 (5)	0.02 (5)	0.02 (5)
		Line2	0.02 (5)	0.02 (5)	0.02 (5)
		Line3	NS	0.02 (5)	0.02 (5)
		Line4	0.02 (5)	0.02 (5)	NS
		Random Selection	NS	NS	NS
Salt	Group	Lines	Generation 3	Generation 4	Generation 6
16S Species evenness	Random Selection Control	Line1	0.04 (4)	NS	NS
		Line2	0.02 (5)	0.02 (5)	NS
		Line3	NS	0.04 (4)	NS
		Line4	NS	NS	NS
		Add-back Control	NS	NS	0.04 (4)
	Add-back Control	Line1	NS	0.02 (5)	0.02 (5)
		Line2	0.02 (5)	0.02 (5)	0.04 (4)
		Line3	NS	0.02 (5)	0.02 (5)
		Line4	NS	0.02 (5)	NS
		Random Selection	NS	NS	NS
Salt	Group	Lines	Generation 3	Generation 4	Generation 6
16S Species richness	Random Selection Control	Line1	0.02 (5)	NA	NA
		Line2	NS	NA	NA
		Line3	NS	NA	NA
		Line4	NS	NA	NA
		Add-back Control	0.02 (5)	NA	NA
	Add-back Control	Line1	0.02 (5)	NA	NA
		Line2	0.02 (5)	NA	NA
		Line3	0.02 (5)	NA	NA
		Line4	0.02 (5)	NA	NA
		Random Selection	0.02 (5)	NA	NA

Table S6 ITS alpha diversity comparisons for the significantly different Generations in the No Salt treatment. All comparisons are 1 degree of freedom. NS is no significance and NA is not applicable as no overall significance was identified. Data is shown as p-value (H-value). Only those Generations with a significant overall difference between all groups are shown. Only comparisons relative to selection and the add-back control are shown.

No Salt	Group	Lines	Generation 2	Generation 5	Generation 6
ITS Species diversity	Random Selection Control	Line1	NS	NS	NS
		Line2	NS	NS	0.02 (5)
		Line3	NS	NS	NS
		Line4	0.02 (5)	NS	NS
		Add-back Control	NS	0.02 (5)	0.04 (4)
	Add-back Control	Line1	NS	0.04 (4)	0.04 (4)
		Line2	0.04 (4)	0.02 (5)	0.02 (5)
		Line3	NS	0.02 (5)	0.04 (4)
		Line4	0.02 (5)	0.02 (5)	0.02 (5)
		Random Selection	NS	0.02 (5)	0.04 (4)
No salt	Group	Lines	Generation 2	Generation 5	Generation 6
ITS Species evenness	Random Selection Control	Line1	NS	NS	NS
		Line2	NS	NS	0.02 (5)
		Line3	NS	NS	NS
		Line4	NS	NS	NS
		Add-back Control	NS	0.02 (5)	NS
	Add-back Control	Line1	NS	NS	NS
		Line2	0.02 (5)	0.02 (5)	0.02 (5)
		Line3	0.04 (4)	0.02 (5)	0.04 (4)
		Line4	0.04 (4)	0.02 (5)	0.02 (5)
		Random Selection	NS	0.02 (5)	NS
No salt	Group	Lines	Generation 2	Generation 5	Generation 6
ITS Species richness	Random Selection Control	Line1	NA	NA	NA
		Line2	NA	NA	NA
		Line3	NA	NA	NA
		Line4	NA	NA	NA
		Add-back Control	NA	NA	NA
	Add-back Control	Line1	NA	NA	NA
		Line2	NA	NA	NA
		Line3	NA	NA	NA
		Line4	NA	NA	NA
		Random Selection	NA	NA	NA

Table S7 ITS alpha diversity comparisons for the significantly different Generations in the Salt treatment. All comparisons are 1 degree of freedom. NS is no significance and NA is not applicable as no overall significance was identified. Data is shown as p-value (H-value). Only those Generations with a significant overall difference between all groups are shown. Only comparisons relative to selection and the add-back control are shown.

Salt	Group	Lines	Generation 4	Generation 5
ITS Species diversity	Random Selection Control	Line1	NS	0.02 (5)
		Line2	NS	0.02 (5)
		Line3	NS	0.04 (4)
		Line4	0.02 (5)	NS
		Add-back Control	NS	0.02 (5)
	Add-back Control	Line1	NS	0.04 (4)
		Line2	NS	0.04 (4)
		Line3	NS	0.02 (5)
		Line4	0.02 (5)	0.02 (5)
		Random Selection	NS	0.02 (5)
Salt	Group	Lines	Generation 4	Generation 5
ITS Species evenness	Random Selection Control	Line1	0.02 (5)	0.04 (4)
		Line2	NS	NS
		Line3	NS	NS
		Line4	0.02 (5)	NS
		Add-back Control	NS	0.04 (4)
	Add-back Control	Line1	NS	NS
		Line2	NS	NS
		Line3	NS	NS
		Line4	NS	0.02 (5)
		Random Selection	0.02 (5)	NS
Salt	Group	Lines	Generation 4	Generation 5
ITS Species richness	Random Selection Control	Line1	NA	NS
		Line2	NA	NS
		Line3	NA	NS
		Line4	NA	0.02 (5)
		Add-back Control	NA	NS
	Add-back Control	Line1	NA	NS
		Line2	NA	0.02 (5)
		Line3	NA	0.02 (5)
		Line4	NA	0.02 (5)
		Random Selection	NA	NS

Table S8 Overall beta-diversity comparisons for bacterial and fungal composition at the OTU level based on PERMANOVA. "Line" refers to Phenotype-Selected Lines, the Random Selection Control, and the Add-back Control.

Gene	Factor	PERMANOVA result		
Bacterial 16S rRNA	Salt	$F_{1,287} = 129$	$R^2 = 0.31$	$p \leq 0.001$
	Generation	$F_{5,282} = 11.5$	$R^2 = 0.17$	$p \leq 0.001$
	Line	$F_{5,282} = 2.7$	$R^2 = 0.05$	$p \leq 0.001$
Fungal ITS	Salt	$F_{1,287} = 41.4$	$R^2 = 0.13$	$p \leq 0.001$
	Generation	$F_{5,282} = 23.5$	$R^2 = 0.29$	$p \leq 0.001$
	Line	$F_{5,282} = 4.3$	$R^2 = 0.07$	$p \leq 0.001$

Table S9 Overall beta diversity comparisons for individual generations. Statistical result was based on a PERMANOVA. All degrees of freedom were (5,18). Overall refers to the overall comparison between the Phenotype-Selected Lines, the Random Selection Control, and the Add-back Control. Detailed comparisons are viewable in Supporting Information Tables S10 and S11.

	No Salt	Generation 1	Generation 2	Generation 3	Generation 4	Generation 5	Generation 6
16S	Overall	$R^2 = 0.35$, $p = 0.02$	$R^2 = 0.33$, $p \leq 0.001$	$R^2 = 0.37$, $p \leq 0.001$	$R^2 = 0.49$, $p \leq 0.001$	$R^2 = 0.46$, $p \leq 0.001$	$R^2 = 0.49$, $p \leq 0.001$
	Salt	Generation 1	Generation 2	Generation 3	Generation 4	Generation 5	Generation 6
16S	Overall	NS	$R^2 = 0.40$, $p \leq 0.001$	$R^2 = 0.50$, $p \leq 0.001$	$R^2 = 0.57$, $p \leq 0.001$	$R^2 = 0.51$, $p \leq 0.001$	$R^2 = 0.62$, $p \leq 0.001$
	No Salt	Generation 1	Generation 2	Generation 3	Generation 4	Generation 5	Generation 6
ITS	Overall	NS	$R^2 = 0.34$, $p \leq 0.001$	$R^2 = 0.42$, $p \leq 0.001$	$R^2 = 0.54$, $p \leq 0.001$	$R^2 = 0.53$, $p \leq 0.001$	$R^2 = 0.69$, $p \leq 0.001$
	Salt	Generation 1	Generation 2	Generation 3	Generation 4	Generation 5	Generation 6
ITS	Overall	NS	$R^2 = 0.26$, $p = 0.04$	$R^2 = 0.34$, $p = 0.004$	$R^2 = 0.53$, $p \leq 0.001$	$R^2 = 0.64$, $p \leq 0.001$	$R^2 = 0.62$, $p \leq 0.001$

Table S10 Bacterial composition beta diversity comparisons. All degrees of freedom were (1,6). NS is no significance. Data is shown as p-value (R2 value). Only PERMANOVA comparisons relative to selection and the add-back control are shown.

No Salt	Lines	Generation 1	Generation 2	Generation 3	Generation 4	Generation 5	Generation 6
Random Selection Control	Line1	0.03 (0.23)	0.05 (0.20)	0.03 (0.28)	0.03 (0.22)	0.03 (0.26)	0.03 (0.24)
	Line2	0.03 (0.17)	0.03 (0.20)	0.04 (0.22)	0.03 (0.22)	0.03 (0.24)	0.03 (0.23)
	Line3	NS	0.03 (0.18)	0.03 (0.20)	0.03 (0.21)	0.03 (0.20)	0.02 (0.22)
	Line4	NS	NS	NS	0.03 (0.20)	0.03 (0.19)	0.02 (0.26)
	Add-back Control	NS	0.03 (0.29)	0.04 (0.33)	0.03 (0.55)	0.03 (0.55)	0.03 (0.52)
Add-back Control	Line1	0.03 (0.19)	0.03 (0.29)	0.03 (0.30)	0.03 (0.51)	0.03 (0.46)	0.04 (0.50)
	Line2	NS	0.04 (0.36)	0.03 (0.34)	0.04 (0.54)	0.04 (0.50)	0.03 (0.50)
	Line3	NS	0.03 (0.33)	0.02 (0.33)	0.03 (0.55)	0.03 (0.52)	0.03 (0.51)
	Line4	NS	0.03 (0.31)	0.03 (0.30)	0.03 (0.50)	0.02 (0.51)	0.03 (0.50)
	Random Selection	NS	0.03 (0.29)	0.04 (0.33)	0.03 (0.55)	0.03 (0.55)	0.03 (0.52)
Salt	Lines	Generation 1	Generation 2	Generation 3	Generation 4	Generation 5	Generation 6
Random Selection Control	Line1	NS	0.04 (0.22)	0.02 (0.31)	0.03 (0.35)	NS	0.04 (0.46)
	Line2	NS	NS	0.03 (0.38)	0.02 (0.30)	NS	0.02 (0.38)
	Line3	NS	NS	0.02 (0.28)	0.03 (0.39)	0.03 (0.28)	NS
	Line4	NS	NS	0.03 (0.37)	0.03 (0.39)	0.03 (0.28)	NS
	Add-back Control	NS	NS	0.04 (0.45)	0.03 (0.55)	0.04 (0.51)	0.03 (0.53)
Add-back Control	Line1	NS	0.03 (0.40)	0.03 (0.39)	0.04 (0.57)	0.02 (0.46)	0.02 (0.63)
	Line2	NS	0.02 (0.40)	0.04 (0.53)	0.03 (0.55)	0.03 (0.48)	0.03 (0.65)
	Line3	NS	0.03 (0.43)	0.03 (0.41)	0.03 (0.55)	0.03 (0.50)	0.03 (0.59)
	Line4	NS	0.04 (0.42)	0.02 (0.51)	0.02 (0.56)	0.03 (0.54)	0.03 (0.62)
	Random Selection	NS	NS	0.04 (0.45)	0.03 (0.55)	0.04 (0.51)	0.03 (0.53)

Table S11 Fungal composition beta diversity comparisons. All degrees of freedom were (1,6). NS is no significance. Data is shown as p-value (R2 value). Only PERMANOVA comparisons relative to selection and the add-back control are shown.

No Salt	Lines	Generation 1	Generation 2	Generation 3	Generation 4	Generation 5	Generation 6
Random Selection Control	Line1	NS	NS	0.04 (0.25)	NS	NS	0.03 (0.33)
	Line2	NS	NS	NS	NS	NS	0.02 (0.67)
	Line3	NS	NS	NS	0.02 (0.33)	NS	NS
	Line4	NS	NS	NS	NS	NS	NS
	Add-back Control	NS	0.03 (0.31)	0.02 (0.46)	0.02 (0.49)	0.03 (0.71)	0.03 (0.62)
Add-back Control	Line1	NS	0.04 (0.38)	0.03 (0.38)	0.03 (0.57)	0.03 (0.52)	0.02 (0.61)
	Line2	NS	0.04 (0.44)	0.04 (0.50)	0.03 (0.56)	0.03 (0.63)	0.03 (0.75)
	Line3	NS	0.04 (0.47)	0.04 (0.57)	0.03 (0.54)	0.03 (0.57)	0.03 (0.65)
	Line4	NS	0.03 (0.27)	0.04 (0.37)	0.04 (0.50)	0.03 (0.63)	0.03 (0.64)
	Random Selection	NS	0.03 (0.31)	0.02 (0.46)	0.02 (0.49)	0.03 (0.71)	0.03 (0.62)
Salt	Lines	Generation 1	Generation 2	Generation 3	Generation 4	Generation 5	Generation 6
Random Selection Control	Line1	NS	NS	NS	0.04 (0.32)	0.02 (0.35)	0.03 (0.30)
	Line2	NS	NS	NS	NS	0.03 (0.43)	0.04 (0.30)
	Line3	NS	0.05 (0.18)	NS	NS	NS	NS
	Line4	NS	NS	NS	NS	NS	0.03 (0.40)
	Add-back Control	NS	0.04 (0.23)	0.04 (0.26)	0.03 (0.52)	0.03 (0.51)	0.03 (0.44)
Add-back Control	Line1	NS	NS	NS	0.03 (0.46)	0.03 (0.68)	0.03 (0.64)
	Line2	NS	0.03 (0.27)	NS	0.03 (0.37)	0.03 (0.51)	0.04 (0.62)
	Line3	NS	0.04 (0.32)	0.03 (0.25)	0.03 (0.50)	0.03 (0.60)	0.04 (0.56)
	Line4	NS	0.03 (0.25)	0.03 (0.36)	0.02 (0.55)	0.02 (0.65)	0.02 (0.66)
	Random Selection	NS	0.04 (0.23)	0.04 (0.26)	0.03 (0.52)	0.03 (0.51)	0.03 (0.44)

Table S12 Comparison of Bray Curtis dissimilarities relative to the Add-back Control between Generations. NS is no significance. All comparisons were 1 degree of freedom. Shown data is p-value (H-value).

Gene	Treatment	Generation 1 vs. Generation 2	Generation 2 vs. Generation 3	Generation 3 vs. Generation 4	Generation 4 vs. Generation 5	Generation 5 vs. Generation 6
Bacterial 16S	No salt	<0.001 (88)	NS	<0.001 (112)	NS	0.002 (10)
	Salt	<0.001 (99)	0.05 (4)	<0.001 (52)	NS	<0.001 (12)
Fungal ITS	No salt	<0.001 (59)	0.05 (4)	<0.001 (78)	<0.001 (71)	<0.001 (47)
	Salt	0.01 (6)	<0.001 (18)	<0.001 (58)	NS	<0.001 (115)

Table S13 Bacteria that are successively selected by the Phenotype-Selected Lines. Data were generated by differential abundance testing in DESeq2 of G3 to G6. Positive fold change means that OTU had a greater relative abundance in the Phenotype-Selected Lines.

Salt	OTU #	Taxa	Log2 fold change	p-adjusted
No Salt	OTU2618355881	Unclassified Holophagaceae	3.27	< 0.001
	OTU9646563467	Dyella japonica	2.82	< 0.001
	OTU4087114411	Unclassified Methylophilaceae	2.53	< 0.001
	OTU9979588299	Unclassified Gemm1	2.33	0.001
	OTU8498993691	Unclassified Sphingomonadaceae	2.19	0.001
	OTU7870397711	Unclassified Ellin6075	2.05	0.005
	OTU5037641516	Unclassified Perlucidibaca	2.04	0.004
	OTU681578660	Unclassified Cytophagaceae	2.00	0.006
	OTU4538863367	Unclassified Comamonadaceae	1.98	0.023
	OTU9570675322	Unclassified Perlucidibaca	1.80	0.020
	OTU4672847499	Unclassified Chlorobi	1.77	0.025
	OTU8780918064	Unclassified Thermomonas	1.74	0.026
	OTU9215546921	Unclassified Devosia	1.08	0.042
Salt	OTU4342871350	Unclassified Flavobacteriaceae	4.12	< 0.001
	OTU9901379637	Unclassified Aquicella	3.91	< 0.001
	OTU2633391341	Unclassified Hyphomonadaceae	3.46	< 0.001
	OTU8151834470	Unclassified Proteobacteria	2.74	< 0.001
	OTU3530057809	Unclassified Cytophagaceae	2.71	< 0.001
	OTU1694599522	Unclassified Flavobacteriaceae	2.60	< 0.001
	OTU438250021	Unclassified Erythrobacteraceae	2.37	0.003
	OTU4900919577	Unclassified Flavisolibacter	2.12	0.001
	OTU3210496264	Unclassified Rhodobacteraceae	2.05	< 0.001
	OTU6734044900	Unclassified Gemmatimonadetes	1.94	0.006
	OTU7616448361	Unclassified auto67_4W	1.92	0.003
	OTU2581698912	Unclassified Planctomyces	1.89	0.006
	OTU714154316	Unclassified Aquicella	1.88	0.008
	OTU9336256758	Unclassified Denitrobacter	1.83	0.004
	OTU6024378526	Unclassified Phyllobacteriaceae	1.62	0.027
	OTU6007375891	Unclassified Rhodoplanes	1.53	0.030
	OTU8510592504	Unclassified Sediminibacterium	1.42	0.044
	OTU9691109341	Unclassified Chitinophagaceae	1.38	0.009
	OTU204949949	Unclassified Erythrobacteraceae	1.30	0.016

Table S14 Bacteria that are successively filtered by the Phenotype-Selected Lines. Data were generated by differential abundance testing in DESeq2 of G3 to G6. Negative fold change means that OTU had a greater relative abundance in the Add-back Control.

Salt	OTU #	Taxa	Log2 fold change	p-adjusted
No Salt	OTU7846824063	Unclassified Ellin6075	-1.69	0.025
	OTU1153084454	Unclassified Flavisolibacter	-1.74	0.005
	OTU4696802307	Unclassified Chitinophaga	-1.79	0.039
	OTU6720208937	Unclassified Niastella	-1.87	0.006
	OTU4875185652	Chitinophaga arvensicola	-1.97	< 0.001
	OTU6810612724	Bacillus flexus	-1.99	< 0.001
	OTU2386211038	Unclassified Rhodanobacter	-2.74	< 0.001
Salt	OTU9989734958	Unclassified Alcanivorax	-0.49	0.020
	OTU6810612724	Bacillus flexus	-1.91	0.027
	OTU4047793499	Unclassified Ramlibacter	-2.30	0.002
	OTU766190230	Unclassified Chitinophagaceae	-2.35	< 0.001
	OTU4012201815	Unclassified Rhodanobacter	-2.68	0.006
	OTU4875185652	Chitinophaga arvensicola	-2.94	0.001
	OTU6994246783	Unclassified Rhodanobacter	-2.94	0.009
	OTU6616956515	Streptomyces lanatus	-3.18	0.002
	OTU4318158338	Unclassified Rhodanobacter	-3.90	< 0.001
	OTU8974348846	Unclassified TM73	-4.02	< 0.001

Table S15 Fungi that are successively selected by the Phenotype-Selected Lines. Data were generated by differential abundance testing in DESeq2 of G3 to G6. Positive fold change means that OTU had a greater relative abundance in the Phenotype-Selected Lines. Negative fold change means that OTU had a greater relative abundance in the Add-back Control.

Salt	OTU #	Taxa	Log2 fold change	p-adjusted
No Salt	OTU335814488	Unclassified Ascomycota	3.98905046	< 0.001
	OTU344319483	Unclassified Trichoderma	1.97112283	< 0.001
	OTU426772175	Mortierella camargensis	1.4896146	< 0.001
	OTU155872480	Unclassified Fusarium	-1.1815699	0.010
	OTU18256744	Unidentified Paraphaeosphaeria	-1.2223921	0.017
	OTU589487257	Unclassified Nectriaceae	-1.2267707	0.004
	OTU404586396	Unclassified Leotiomycetes	-1.285402	0.004
	OTU983336604	Unidentified Blastobotrys	-1.3752113	< 0.001
	OTU154537978	Cryptococcus terreus	-1.591915	< 0.001
	OTU333080995	Monographella cucumerina	-1.6988302	< 0.001
	OTU470186797	Unclassified Nectriaceae	-1.7705178	< 0.001
	OTU917859397	Unclassified Leotiomycetes	-1.7849869	< 0.001
	OTU398406512	Unclassified Penicillium	-2.6988302	< 0.001
Salt	OTU60114369	Unclassified Sordariales	4.47	< 0.001
	OTU335814488	Unclassified Ascomycota	2.20	0.001
	OTU344319483	Unclassified Trichoderma	1.96	< 0.001
	OTU255177263	Candida subhashii	1.73	< 0.001
	OTU485732059	Candida subhashii	1.19	0.001
	OTU968017296	Mucor circinelloides	1.17	0.023
	OTU741659813	Candida subhashii	1.13	0.001
	OTU586490526	Penicillium lapidosum	-0.89	0.023
	OTU155872480	Unclassified Fusarium	-1.06	< 0.001
	OTU589487257	Unclassified Nectriaceae	-1.18	< 0.001
	OTU404586396	Unclassified Leotiomycetes	-1.90	< 0.001
	OTU917859397	Unclassified Leotiomycetes	-1.93	< 0.001
	OTU398406512	Unclassified Penicillium	-2.09	< 0.001
	OTU18256744	Unidentified Paraphaeosphaeria	-2.66	< 0.001

Table S16 Microbial genera that are consistently selected or filtered over time by the Phenotype-Selected Lines. Data were generated using a network analysis from the Phylosmith package. Only those taxa with a consistent statistical association with every Generation were selected. Data are shown as: p-value (Spearmans rho) for each Generation.

Gene	Salt treatment	Taxa name	G2	G3	G4	G5	G6
16S rRNA	No salt	Pseudoxanthomonas	< 0.001 (0.4)	0.02 (0.3)	0.04 (-0.2)	0.04 (-0.2)	0.04 (-0.2)
		Salinibacterium	< 0.001 (-0.6)	0.001 (-0.4)	< 0.001 (0.4)	0.02 (0.26)	0.007 (0.3)
		Asticcacaulis	< 0.001 (-0.4)	0.04 (-0.2)	0.04 (-0.2)	0.004 (0.3)	< 0.001 (0.6)
	Salt	Dyella	< 0.001 (0.7)	0.01 (0.3)	0.004 (-0.3)	< 0.001 (-0.4)	0.02 (-0.25)
ITS	No salt	Blastobotrys*	N/A	< 0.001 (0.5)	0.002 (0.3)	0.03 (-0.2)	< 0.001 (-0.6)
	Salt	Geomyces	< 0.001 (0.6)	0.03 (0.2)	0.006 (-0.3)	0.03 (-0.2)	0.006 (-0.3)

*Taxa was significantly associated in all Generations except G2
